# Evaluation of Time Management Behaviors and Its Related Factors in the Senior Nurse Managers, Kermanshah-Iran

**DOI:** 10.5539/gjhs.v7n2p366

**Published:** 2015-01-20

**Authors:** Arash Ziapour, Alireza Khatony, Faranak Jafari, Neda Kianipour

**Affiliations:** 1 Kermanshah University of Medical Sciences, Kermanshah, Iran; 2 Social Development and Health Promotion Research Center, Kermanshah University of Medical Sciences, Kermanshah, Iran; 3 Kermanshah School of Nursing and Midwifery, Kermanshah University of Medical Sciences, Kermanshah, Iran; 4 Department of Environmental Health Engineering, Kermanshah University of medical sciences, Kermanshah, Iran

**Keywords:** evaluation, nurse managers, time management behaviors

## Abstract

**Background and Objective:**

Time management is an extensive concept that is associated with promoting the performance of managers. The present study was carried out to investigate the time management behaviors along with its related factors among senior nurse mangers.

**Materials and Methods:**

In this descriptive-analytical study, 180 senior nurse managers were selected using census method. The instrument for data collection was a standard time behavior questionnaire. Data were analyzed by descriptive and analytical statistics.

**Results:**

The findings showed that among the dimensions of time management behaviors, setting objectives and prioritization, and mechanics of time management dimensions obtained the highest and lowest frequency, respectively. Comparison of the mean scores of time management behaviors indicated a significant difference in the gender (p<0.05), age (p<0.001), education (p=0.015), job experience (p<0.001), managerial experience (p<0.001) and management rank management (p<0.029).

**Conclusion:**

On the whole, senior nurse managers enjoyed a favorable time management skill. Given the importance of time management behaviors, it seems that teaching these behaviors more seriously through regular educational programs can effectively promote the performance of senior nurse managers.

## 1. Introduction

Time management is an extensive concept in various executive and managerial domains and is related to the promotion of the managers’ qualitative performance (Macan, 2010). Time management is the best method of using the time to achieve the personal and occupational objectives, that creates a direct relationship between performing daily activities, on the one hand, and ensuring job satisfaction and progress, on the other hand (Edwin, 2004). Frost considers time as a pressure factor everywhere and argues that time management is an efficient tool to cope with this problem (Brigitte & Christel, 2007).

Time management techniques promote job performance, provide more time to carry out the tasks with higher priority and accelerate the activities (Thomack, 2012; Belal Said, 2014; Baghdasariyan et al., 2011), thereby, enhancing the individuals’ job satisfaction (Kaya et al., 2012).

Management and leadership are vital phenomena in all domains of social activities (Leonidas et al., 2010). Human and material resources will be destroyed in the absence of qualified management and leadership. However, time mnagement in the realm of health and for nurse managers is of paramount importance (Mirzaei et al., 2012) because it is directly related to the people’s health, and waste of time and lack of time management in the domain of nurse managers cause a reduction in efficiency (Soleimani et al., 2011).

Several factors including planning skills, organization, administration and control affect time management behaviors in the realm of health (Khadam & Kelagari, 2009; Ghorbanshiroudi et al., 2011; Belal Said, 2014; Grissom et al., 2013; Rosario, 2012). Numerous studies have been conducted on time management behaviors among managers. Mohammadian et al (2006) reported that time management skill was at a very good level among 15.4% of managers, being higher in female managers than in the male counterpart. The results of another study indicated that time management reduced job stress among nurses (Hashemizadeh, 2006; Gorbanshiroudi et al., 2011). In their study, Kearns and Gardiner (2007), Stwart & Lombard (2007) and Claessensal (2007) stated that making use of time management had a positive effect on such outcomes as academic performance, academic achievement, problem solving skill and reduction of physical tensions and stress.

The findings of Hemmatian (2009) showed a significant correlation between the managers’ time management and their managerial experience and age. Brigitte & Christel (2007) reported a positive relationship between time management and job satisfaction. Leonaidse (2010) reported a significant correlation between time management and the staff’s creativity. Also, Hashemizadeh (2006) argued that time management behaviors decrease job stress in nurses.

Given the significance of this issue and lack of knowledge about the factors affecting time management behaviors among senior nurse managers at Kermanshah University of Medical Sciences, the present study was aimed to analyze the given factors. The results of the present study are hoped to be a step taken to promote time management behaviors among nurse managers and consequently to enhance organizational efficiency.

## 2. Materials and Methods

This descriptive-analytical study was carried out at hospitals affiliated to Kermanshah University of Medical Sciences in 2014. The sample consisted of 180 senior nurse managers (16 matrons, 57 supervisors and 97 head nurses) working in different wards in the 6 hospitals affiliated to Kermanshah University of Medical Sciences. All of these hospitals are general with 3129 active beds and 1987 nurses. Given the limited number of senior nurse managers at Kermanshah University of Medical Sciences, all the participants were included in the study via census method. The inclusion criteria comprised of having at least bachelor degree in nursing, working in one of the nursing managerial positions and having minimum 6 months experience in the current position.

The instrument for data collection was a researcher made questionnaire consisting of two sections. The first section contained 6 items about demographic information such as gender, age, education, work experience, managerial experience and managerial ranking. The second section was about time management behaviors which included 32 items. It comprised of four domains, 8 items in each domain. The given domains were “setting objectives and prioritization”, “mechanics of time management”, “control over time” and “maintaining order and organization” (Hashemizadeh, 2006).

To determine the validity of the questionnaire, the content validity measure was applied. To this end, the questionnaire was given to 12 faculty members, and their comments were considered. The reliability of the questionnaire was confirmed by Cronbach’s alpha (r=0.93).

The items of time management behavior questionnaire were designed based on 5-point Likert scale, each item with 5 responses, including completely disagree, disagree, no idea, agree and completely agree, scored from 1 to 5, respectively. The respondents selected one response for each item as a criterion compatible with themselves. The total range of the questionnaire was 25-125 with a higher score indicating higher time management behavior. The levels of time management behaviors were classified as very little skill=0-24, little skill=25-49, average skill=50-74, good skill=75-99 and very good skill=75-100.

To carry out the study, first, written permission was taken from deputy of research and technology of the given university and presented to the authorities of hospitals affiliated to Kermanshah University of Medical Sciences. Then, the names of the nurse managers along with their weekly schedule were taken from the nursing office in each hospital. Next, the researcher visited the participants in their working hours to complete the questionnaires. The objectives of the study were explained to the participants, confidentiality of the obtained information, including personal characteristics of the respondents and their responses, was confirmed and informed consent was taken from the samples.

The questionnaires were completed by the samples and collected by the researcher. Data were analyzed by SPSS-18 software using descriptive statistics (frequency (%), mean and standard deviation) and analytical statistics (one-way ANOVA and independent t-test). Independent t-test was used to compare the scores of time management behaviors in terms of nominal qualitative variables like gender, and one-way analysis of variance (ANOVA) was applied to compare the scores of time management behaviors in terms of ranked quantitative variables (age, education, work experience, managerial experience) and multilevel qualitative variables (managerial rank). P<0.05 was considered statistically significant.

## 3. Results

From the total 180 senior nurse managers at the hospitals affiliated with Kermanshah University of Medical Science, 68 (37.8%) managers were male and 112 (62.2%) of them were managers were female. Approximately half of the samples (n=86, 47.8%) were in the age range of 31-40 (Mean±SD=32.7±6.8). In terms of education, 105 (58.3%) managers had bachelor degree and 87 (48.3%) of them had 6-10 years of work experience (Mean±SD=8.7±5.4). With regard to managerial rank, 97 (53,9%) samples were head nurses and 67 (37.2%) of them were supervisors. About half of the samples (n=92, 51.1%) had 6-10 years of managerial experience (Mean±SD= 8.6±4.9).

The results showed that the majority of the units under study (96.7%) were at a favorable level in terms of time management behaviors (Mean±SD=4.23±0.35). The highest level of time management behaviors was reported for setting objectives and prioritization (Mean±SD=4.33±0.4), followed by control over time (Mean±SD=4.25±0.59), order and organization (Mean±SD=4.24±0.43) and mechanics of time management (Mean±SD=4.13±0.46).

Further, the findings indicated a statistically significant difference between males and females in terms of time management behaviors, with males showing more time management behaviors (mean=4.35 vs 4.14, t=3.800, p<0.05). Significant difference, however, was found between time management behaviors and demographic variables (age, education, work experience, managerial experience and managerial rank) (Table 2).

**Table 1:** Demographic Characteristics of participants

Variables	Groups	No(%)
Sex	male	68(37.8)
	female	112(62.2)
	
Age(years)	≥30	73(40.6)
	31-40	86(47.8)
	41-50	14(7.8)
	≤51	7(3.9)
	
Education	Bachelor’s degree	105(58.3)
	Master’s Degree	75(41.7)
	
Job Experience(years)	≥5	54(30)
	6-10	87(48.3)
	11-15	19(10.6)
	16-20	13(7.2)
	≤21	7(3.9)
	≥5	39(21.7)
	
Management Experience(years)	6-10	92(51.1)
	11-15	42(23.3)
	≤16	7(3.9)
	
Management Order	nurse managers	16(8.9)
	Super Visor	67(37.2)
	Head Nurse	97(53.9)

**Table 2:** Comparison of the mean and standard deviation of time management scores according to the samples’ characteristics

variables	groups	setting objectives and prioritization		mechanics of time management		control over time		maintaining order and organization		Overall	

		Mean	*pv*	Mean	*pv*	Mean	*pv*	Mean	*pv*	Mean	*pv*
											
**Age(years)**	≥30	4.23	< .05*	3.96	<0.05*	4.35	<0.05*	4.18	< 0.05*	4.18	<0.001*
	31-40	4.40		4.32		4.26		4.28		4.31	
	41-50	4.20		3.70		3.50		4.10		3.87	
	≥51	4.80		4.40		4.40		4.60		4.55	
	
**Education**	BSc	4.29	<0.05*	4.02	< 0.05*	4.27	<0.05*	4.23	< 0.05*	4.20	0.015*
	MSc	4.38		4.28		4.20		4.25		4.28	
	
**Job experience**	5≥	4.11	<0.05*	3.91	0.11	4.42	<0.05*	4.15	< 0.05*	4.15	<0.001*
	6-10	4.43		4.34		4.32		4.28		4.34	
	11-15	4.46		3.71		3.69		4.20		4.01	
	16-20	4.09		4.09		3.72		4.18		4.02	
	≥51	4.80		4.40		4.40		4.60		4.55	
	
**Managerial experience**	5≥	4.57	<0.05*	4.67	<0.05*	4.37	<0.05*	4.35	< 0.05*	4.49	<0.001*
	6-10	4.31		3.95		3.98		4.22		4.12	
	11-15	4.05		3.97		4.65		4.11		4.20	
	≥16	4.80		4.40		4.40		4.60		4.55	
	
**Management order**	nurse managers	4.45	<0.05*	4.23	<0.05*	4.06	<0.05*	4.50	< 0.05*	4.31	<0.029*
	Super Visor	4.21		3.97		4.26		4.13		4.15	
	Head Nurse	4.39		4.22		4.26		4.27		4.28	

* Statistically significant.

The results obtained regarding the age showed that the age groups of >50 and 41-49 years had the highest and lowest time management behaviors, respectively (f=4.530, p<0.001). With regard to education, the findings showed that the managers with master and bachelor degrees had the highest and lowest levels of time management behaviors, respectively (f=3.046, p=0.015). Further, the results revealed that managers with work experience of >21 and 11-15 years had the maximum and minimum levels of time managemnet behaviors, respectively (f=9.117, p<0.001) and managers with managerial experience of >21 and 6-10 years had the highest and lowest levels of time management behaviors, respectively (f=16.434, p<0.001). Moreover, the maximum and minimum levels of time management behaviors were reported for nurse managers and head nurses, respectively (mean=4.23 vs 4.22, p<0.029).

## 4. Discussion

The present study was conducted to investigate the time management behaviors and the factors related to it among the senior nurse managers at Kermanshah University of Medical Sciences. Generally, senior nurse managers possessed a favorable level of time management skill. The results of Mohammadian et al. (2006) showed that 57.7% of managers were at a higher than average level in terms of time management behaviors. The findings of the study by Hosseini et al. (2014) demonstrated that the head nurses and nurses of the hospitals affiliated with social security office obtained a high level of time management behaviors. The results of Hasumi and Sarikhani’s (2010) study indicated ≥ average level of time management behaviors for the personnel of Azad university. The results of the study by Kaya et al. (2012) carried out on nursing and midwifery students in Istanbul University indicated that 63.4% of them had good time management skills. The findings of the present research revealed that nurse managers had a good command of time management skill. It can be concluded that the work objectives of the senior nurse managers have always been definite and are classified according to the prioritization system.

Moreover, the findings showed that time management skill was significantly higher in females than in males. The results of the present study are in line with the findings of Liu et al (2009), Alam et al. (2013), Mazaheri and Eivazzadeh (2012) and Karami Moghadam (1998). However, the results of the studies of Macan et al. (2010) and Guoging & Yongxin (2000) indicated lower levels of time management skill among women than men. In contrast, Sheikh Nezami (1997), Saketi and Taheri (2010), Guoqing (2000) and Jahansir et al (2007) reported no role for gender in time management. Various results have been reported by the previous studies, some of which are in line with the findings obtained in the present study. However, time management is an acquired feature that is nurtured through education and is not highly associated with gender.

In the current study, the managers with master’s degree had the highest level of time management skill, which is in agreement with the findings of the studies performed by Di (1996), Ravari et al (2008), Ling et al (2006) and Hosseini et al (2010). The authors tend to think that capabilities of the nurse managers in time management is enhanced with an increase in their education level.

In the current study, time management skill in various age groups was significantly different and >50 year-old managers had the highest level of time management skill. The findings of the previous studies are alos indicative of a direct correlation between age and time management (Abdolvand et al., 2010; Hemmatian, 2009), which are in line with the results of the present study. The capabilities of the managers in terms of time management are expected to rise with age increase.

The managers with >21 years of work experience had a higher time management skill. The studies carried out in this regard also indicate a significant correlation between work experience and time management skill (Hemmatian, 2009; Khadam, & Kelagari, 2009 and Bagheri & UsfiNezhad Atari, 2012). The authors of the present study believe that time management skill is enhanced among the nurse managers with an increase in their work experience.

With regard to various domains of time management, the findings showed that senior nurse managers had the highest level of skill in “setting objectives and prioritization” domian. Studies have also shown that nurses make use of various strategies for time management, the most important of which is setting objectives and prioritization (Zampetakis et al., 2010; Waterworth, 2003; Walker, 2004 and Bowser, 2001). Given the critical nature of nursing profession, especially the role of senior nurse managers at hospitals, such a result seems logical and it is evident that the working objectives of senior nurse managers are always classified based on prioritization system.

The results also revealed that the nurse managers possessed good skill about “control over time” domain, which is in contrast with the findings of the studies conducted by Khadam & Kelagari (2009), Lipscomb et al (2010) and Hashemizaseh (2006) which showed control over time was the weakest domain among the dimensions of time mnaagement. The authors believe that control over time is required to be at maximum level; thus, they recommend management retraining programs in which a special heed is given to this domian and the factors wasting the time in the workplace are identified.

In the domain of maintaining order and organization, the nurse managers enjoyed a good skill. Most of the senior nurse managers reported they were adapted to their working conditions and they were able to organize all their occupational and non-occupational issues. In line with this, the findings of Gran-Moravec et al. (2005), Maccan (1996) and Khadam & Kelagari (2009) indicated that organization enhances time management skill in nurses. Having discipline at wok provides a better environment, saves time and consequently improves job performance.

As for the domain of the mechanics of time, the nurse managers possessed a good skill. Most of the samples declared that they had always definite responsibilities in their wards. The results of the present study are in line with the findings of Kaya et al (2012), Ghaedmohammadi (2010), Hashemizadeh (2006) and Khadam & Kelagari (2009). The nurse managers prepare a list of their daily activities to schedule and perform their tasks in the best way possible.

The limitations of this study includepoor generalizability of the results due to different personal, cultural, social and occupational characteristics of the samples as well as data collection procedure which was based on the self-reports, a factor that may have affected the accuracy of the results.

Short-term training programs or training workshops are recommended to be held for senior nurse mangers and the entire nursing staff in order to enhance time management behaviors and skills. Similar studies are also suggested to be carried out on senior nurse managers of other state-run or private hospitals.

## 5. Conclusion

Most of the nurse managers enjoyed a favorable status regarding time management behaviors. Comparison of the mean scores of time mangement behaviors indicated a significant difference in the gender, age, education, job experience, managerial experience and management rank. Given the significance of time management issue, a special attention is needed to be given to time mangement at healthcare centers in Iran because promoting the capabilities of nurse mangers in time mangement behaviors increases the efficiency in healthcare centers. Therefore, selecting and training the nurse managers according to time mangement behaviors can bring about positive outcomes in healthcare centers.
